# Topology Design of Soft Phononic Crystals for Tunable Band Gaps: A Deep Learning Approach

**DOI:** 10.3390/ma18020377

**Published:** 2025-01-15

**Authors:** Jingru Li, Minqi Qian, Jingming Yin, Wei Lin, Zhifu Zhang, Shihao Liu

**Affiliations:** 1 School of Mechanical and Electrical Engineering, Hainan University, Haikou 570228, China; q1961361499@163.com (M.Q.); 22220854060008@hainanu.edu.cn (J.Y.); jeff.zfzhang@foxmail.com (Z.Z.); liushihao1102@126.com (S.L.); 2 Qingdao Innovation and Development Center of Harbin Engineering University, Qingdao 266400, China; linwei23@hrbeu.edu.cn

**Keywords:** intelligent inverse design, deep learning, topology layout, soft phononic crystals, tunable band gaps

## Abstract

The phononic crystals composed of soft materials have received extensive attention owing to the extraordinary behavior when undergoing large deformations, making it possible to provide tunable band gaps actively. However, the inverse designs of them mainly rely on the gradient-driven or gradient-free optimization schemes, which require sensitivity analysis or cause time-consuming, lacking intelligence and flexibility. To this end, a deep learning-based framework composed of a conditional variational autoencoder and multilayer perceptron is proposed to discover the mapping relation from the band gaps to the topology layout applied with prestress. The nonlinear superelastic neo-Hookean model is employed to describe the constitutive characteristics, based on which the band structures are obtained via the transfer matrix method accompanied with Bloch theory. The results show that the proposed data-driven approach can efficiently and rapidly generate multiple candidates applied with predicted prestress. The band gaps are in accord with each other and also consistent with the prescribed targets, verifying the accuracy and flexibility simultaneously. Furthermore, based on the generalization performance, the design space is deeply exploited to obtain desired soft structures whose stop bands are characterized by wider bandwidth, lower location, and enhanced wave attenuation performance.

## 1. Introduction

Phononic crystals (PnCs) [1] or metamaterials (MMs), a typical class of periodic composites, have become one of the hottest research topics in the field of vibration and sound reduction owing to the inherent characteristics of band gaps. In general, they consist of two components; in detail, the component of pass band represents frequency ranges where the wave propagation is allowed, and the component of stop band denotes frequency intervals where the wave propagation is prohibited. The Bragg scattering (BS) [2] and local resonance (LR) [3] are the primary mechanisms for inducing band gaps, based on which increasingly elaborate designs of the composites relating to the geometries, materials, boundaries, and attached mechanisms have been explored to meet various requirements, such as multi-stop bands [4,5,6,7], low-frequency stop bands [8,9,10,11] and broad bandwidth [12,13,14,15]. This facilitates the developments in a wide range of engineering applications, including vibration isolators [16,17,18], wave filters [19,20,21], sound insulation [4,22,23], wave guides [24,25,26], and energy harvesting [27,28,29]. Motivated by the applicability of band gaps, the tunable band structures endowed with more flexibility to manipulate wave propagation and attenuation are expected to gain more significant scientific and technological value. Compared to ordinary materials, soft materials can undergo large deformation when subjected to external mechanical, electrical, magnetic, or thermal loads, leading to adjusting the constitutive behavior in an active manner, which lays a solid foundation for realizing tunable band gaps [30,31,32,33,34,35,36]. Especially, the technique of exerting mechanical prestress or prestretch is a stable tool to measure the external load and has been gradually applied to assemble soft PnCs for regulating wave performance. Based on the nonlinear elasticity theory [37], Huang et al. [38] studied the band structure of one-dimensional (1D) compressible hyperelastic PnCs and validated the efficiency to tune band gaps via applying longitudinal prestress. Afterward the authors [39] included the post-buckling deformations in the design of the PnC plate made of soft materials, where the resonant units were periodically displayed. The results demonstrated remarkable controllability of band gaps with small prestretch or extension that can be realized if strong resonance appears for some particular modes. Wu et al. [40] analyze the axisymmetric guided wave propagation in a pressurized functionally graded (FG) elastomeric hollow cylinder and find that material tailoring along with an adjustment of the pre-stretch and pressure difference can be used to control the propagation of elastic waves. Under the equibiaxial compression, a two-dimensional (2D) acoustic metamaterial was designed by Ning et al. [41], which was composed of resonating elements embedded in the elastomeric matrix. In this study, the numerical results indicated the locally resonant and Bragg scattering band gaps can be simultaneously controlled by the deformation. Wang et al. [42] also applied the soft materials to compose metamaterials for obtaining lower frequency band gaps and demonstrated that the proposed design is able to have a good performance in the design of low-frequency band gaps with large amplitude.

From previous studies, it can be concluded that the tunability of soft PnCs makes them promising candidates as adjustable components of adaptive metamaterials via integrating with other mechanisms, such as the local resonance and inertial amplification mechanisms. As the mentioned mechanisms have been already realized by the applications in practice [43,44,45], the adaptive metamaterials based on the soft PnCs can be further designed to isolate the vibration and noise of the automobile, pipe system, building, ship engineering, and so on. For instance, the soft PnCs can be conceived as the substrates of sandwich structures [46], whose large deformation is able to control the location of the inner cores. Thus, fulfilling the task of solving low-frequency vibration problems of the marine power system using the tunable band gaps. Also, the combination of different layouts of soft PnCs embedded with sophisticated cavities or local resonant elements is helpful in absorbing sound in water, promoting the engineering application in the field of anechoic coating [47]. As can be seen, the adaptive PnCs made of soft materials exhibit great potential and high efficiency in controlling band gaps. Also, intensive dependency can be observed from the external stimuli, material properties, geometrical parameters, and topological layout. Hence, in order to make full use of the improved tunability level, the design space of soft PnCs should be deeply exploited by the method of the inverse design—a one-fits-all approach with the function of offering solutions with desired performances [48]. Using this approach, on-demand designs can be achieved with respect to prescribed tunable band gaps, which is also a main focus of the present work.

The optimization technique is regarded as a prominent tool to perform the task of inverse design. In recent years, considerable efforts have been devoted to searching for the optimal design of PnCs and MMs to acquire target properties of band gaps using gradient-based or gradient-free optimization algorithms. The size optimization [49], the shape optimization [50], and especially the topology optimization (TO) [51,52,53,54,55,56,57,58,59,60], is able to explore the periodic space to a full extent, have drawn intensive interest and been conducted in combination with the band structure analysis methods. Nevertheless, the design space is required to be updated at each iteration during the optimization procedure, which makes the simulations conducted for calculating band gaps recurrent and time-demanding. Also, if the objective is varied, researchers need to restart the entire framework of the optimization project to find the new optimal; that is, an iterative optimization process is required to be completed again. To eliminate the above limitations, the data-driven method that is able to derive the mapping from band gaps to configurations of PnCs in a real-time manner is indispensable. To this end, deep learning (DL), a representative technique of machine learning (ML), which originated from the artificial neural network (ANN), is able to fulfill this task. The term “deep learning” was first proposed by Hinton et al. [61], and remarkable achievements have been obtained in the fields of computer vision [62], natural language processing [63,64], speech recognition [65], decision-making [66], and so on [67] by this flourishing method. In line with the research stream, the DL gradually progressed into the inverse design of artificial structures through two patterns of frameworks: in one approach, the DL technique is integrated with optimization algorithms to optimize the hyperparameters of neural networks instead of manual setting or function as the surrogate model to improve the efficiency of optimal searching [68,69,70,71,72,73]; in another approach, the DL model is established to produce the sophisticated man-made structures directly with respect to given targets [48,74,75,76,77,78]. The latter approach definitely exhibits more flexibility and instantaneity, but owing to the “one-to-many” phenomenon, uncertain problems appear as a result of solving the inverse problems of PnCs and MMs, as different unit cells can share the same band gaps.

To address the above issue, the DL models designed in an unsupervised learning way, such as the tandem neural network (TNN) [79], the autoencoder (AE) [80], and the generative adversarial network (GAN) [81], are proposed to eliminate the negative effects and have been successfully used in generating feasible PnCs to match target features of band gaps. Through training, the TNN composed of multilayer perceptron (MLP), 1D phononic slabs can be obtained automatically within prescribed band gaps [82]. Then Li et al. [83] focused on the flexural wave attenuation at the nanoscale and combined TNN with the probabilistic distribution to complete the inverse design of phononic nanobeams made up of functionally graded materials, where the results demonstrated that the well-trained DL model can provide multiple feasible designs that possess the same man-made band gaps. The MLP was also directly applied to complete the inverse design of structural parameters of the Y-shaped core sandwich beam for given transmission characteristics based on the band gaps, where the average relative error of outputted structural parameters is below 3% [84]. Lee et al. [78] employed the deep neural network (DNN), TNN, conditional variation autoencoder (CVAE), and conditional GAN (CGAN), respectively, to implement the inverse design framework for a one-dimensional defective PnC as a narrow bandpass filter. The diversity performances were compared among them, indicating that the frameworks using the CVAE and the CGAN can present the best performance by solving the nonunique response-to-design mapping problem. When extended to the 2D space, the topological features of in-plane PnCs were extracted and mapped to the distribution of pass bands and stop bands by establishing the AE and MLE models. The topology layouts were generated within anticipated band gaps as a result [85]. In order to offer controllability and stability, Xiao et al. [86] proposed a topology generator based on the CGAN that was applied to generate candidate topological structures with geometric constraints. And then this approach was combined with the automatic differentiation technique to acquire target band gaps. Moreover, the dispersion curves, which determine the boundaries of band gaps, were regarded as inputs and proactively tailored to achieve diverse configurations of 2D PnCs using CGAN [87]. However, to the best knowledge of the authors, the DL-based inverse designs were mainly concentrated on the desired performance that the unit cells made up of linear elastic hard materials can achieve, the intelligent framework responsible for providing feasible design of soft PnCs characterized by topology and applied mechanical load at an instant speed has not been reported so far. Inspired by this fact, the present work aims at establishing the framework of inverse design based on the DL, and the objective is to generate multiple soft PnCs that can induce customized band gaps and efficiently offer designs corresponding to tunable stop bands within lower frequencies, wider bandwidth, or enhanced attenuation levels. It is worth mentioning that, in contrast to the studies using the TO technique to design soft compressible composites where the optimization projects were performed under constant mechanical load [46,88], the present work regards both the discrete and continuous parameters as the design variables. In other words, the design of a soft PnC is represented by its material distribution in conjunction with the applied prestress. The proposed inverse framework is concentrated on presenting multiple feasible solutions concerning the same given input. Further, the generalization and diversity abilities can also make it possible to acquire on-demand designs. In addition, when composing the dataset for training and testing the DL-based model, the present work adopts the transfer matrix method (TMM) rather than the finite element method (FEM) to calculate the tunable band structure, as the former approach is superior in terms of computation efficiency.

The remainder of this paper is organized as follows: Section 2 presents the formulations of wave motion through 1D soft PnC based on the nonlinear elastic theory and the derivations of TMM modeling to predict tunable band gaps. In Section 3, a DL-based framework for the inverse design is established, where the components are delineated to show how accuracy and diversity can be assured. Section 4 gives the generated results of the topological layout with applied prestress, which corresponds to various on-demand requirements of longitudinal wave propagation and attenuation characteristics. Finally, Section 5 summarizes the conclusions reached in this paper.

## 2. Model Descriptions and Theoretical Formulations

This section aims to describe the mechanical properties of the 1D soft PnCs using the neo-Hookean model to illustrate the effects induced by the external prestress and establish the model based on the TMM to obtain the tunable band structures of longitudinal waves, which are shown in Section 2.1 and Section 2.2, respectively.

### 2.1. The Constitutive Properties from Nonlinear Elasticity Theory

In this subsection, the soft materials are assumed to be compressible and homogeneous, and the PnC is composed of two soft phases. Following the nonlinear elasticity theory [89], the initial configuration of the considered structure without any deformation is taken as the reference configuration, where each point is marked by a position vector **X**. When simulated by the external prestrain or prestress, the points on the reference configuration are expected to move to new points, and the position vector on the current configuration is denoted by ***x***. The mapping relationship between ***x*** and **X** is defined as:

(1) x=κ(X,t)

From Equation (1), the deformation gradient **F** can be obtained by F=∂x/∂X, and its determinant *J* = det(**F**) > 0, which is able to determine the local measure of the volume change. Ignoring the dissipative effects of soft compressible elastomers, the first Piola-Kirchhoff stress tensor **P** is acquired by differentiating the energy density function *ψ*(**F**), that is, P=∂ψ(F)/∂F, and the Cauchy stress tensor ***σ*** is related to the tensor **P**: ***σ*** = *J*^−1^**PF**^T^. Further, assuming that there are no mechanical body forces, the mechanical momentum balance equation is written as:

(2) $$\mathrm{Div}(P)=\rho_{0}\partial^{2}x/\partial t^{2}$$

where, *ρ*_0_ denotes the initial density of the material. If performing static or quasi-static calculations, the inertial part can be omitted, and Equation (2) is correspondingly rewritten as: Div(**P**) = 0. Additionally, the ideal compressible neo-Hookean model is employed to derive the free energy density function:

(3) $$\psi (I_{1},I_{3})=(I_{1}-3)\mu /2+(I_{3}^{-\beta }-1)\mu /(2\beta )$$

where *I*_1_ = tr(**C**) (**C** = **F**^T^**F**) and *I*_3_ = det(**C**), which represent the first and third invariants of the right Cauchy strain tensor, respectively. *v* and *μ* denote the Poisson ratio and shear modulus of the elastomer phase, and the parameter *β* is defined as *β* = *ν*/(1−2*ν*). Based on the above expressions, the Cauchy stress tensor ***σ*** is required to satisfy:

(4) $$J\sigma =2FF^{T}\partial \psi /\partial I_{1}+2I_{3}\xi \partial \psi /\partial I_{3}$$

where ***ξ*** is the Kronecker symbol. Considering the infinitesimal increment in the displacement field, the incremental form of the mechanical momentum balance equation is:

(5) $$\mathrm{Div}(\bar{P})=\rho_{0}\partial^{2}\bar{u}/\partial t^{2}$$

In Equation (5), $\bar{P}$ is the incremental change of the first Piola-Kirchhoff stress tensor and $\bar{u}$ is the incremental displacement. The incremental change in the deformation gradient at this time is $\bar{F}=\mathrm{Grad}(\bar{u})$. Through linearizing the constitutive law, the incremental change of the first Piola-Kirchhoff stress tensor is obtained by ${\bar{P}}_{iJ}=C_{0iJkL}{\bar{F}}_{kL}$ and *C*_0*iJkL*_ represent the fourth-order referential elastic moduli tensor, which is determined via *C*_0*iJkL*_ = ∂^2^*ψ*/(∂*F_iJ_*∂*F_kL_*). Substituting this relation into Equation (5) leads to: $C_{0iJkL}\partial^{2}{\bar{u}}_{k}/(\partial X_{J}\partial X_{L})=\rho_{0}\partial^{2}{\bar{u}}_{i}/\partial t^{2}.$ After transforming the reference configuration to the current configuration, it can be further updated as follows:

(6) $$C_{ijkl}\partial^{2}{\bar{u}}_{k}/(\partial x_{j}\partial x_{l})=\rho \partial^{2}{\bar{u}}_{i}/\partial t^{2}$$

where *ρ* = *J*^−1^*ρ*_0_, denotes the density of the material in its current configuration, and *C_ijkl_* = *J*^−1^*C*_0*iJkL*_*F_jJ_F_lL_*, represents the equivalent elastic modulus. According to the neo-Hookean model, the equivalent elastic modulus *C_ijkl_* considering instantaneous effects induced by the static finite deformation, can be obtained by:

(7) $$\begin{array}{c} C_{ijkl}=\mu J^{-1}F_{iJ}F_{kL}(\xi_{jl}\xi_{JL}+I_{3}^{-\beta }F_{Jl}^{-1}F_{Lj}^{-1}+2\beta I_{3}^{-\beta }F_{Jj}^{-1}F_{Ll}^{-1})\\ =\mu J^{-1}(\xi_{jl}F_{iL}F_{kL}+I_{3}^{-\beta }\xi_{il}\xi_{kj}+2\beta I_{3}^{-\beta }\xi_{ij}\xi_{kl}) \end{array}$$

### 2.2. Dispersion Analysis of Tunable Band Structure

The dynamic characteristics of longitudinal waves are examined in this subsection, based on which the formulations to acquire band structures are derived by applying the TMM. This approach is responsible for providing the labels to compose the dataset.

#### 2.2.1. The Governing Equation of Longitudinal Wave

**Figure 1:**
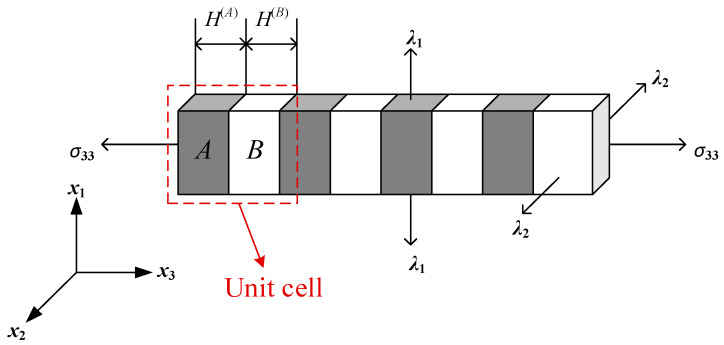
The schematic diagram of finite 1D soft phononic crystals with applied stress *σ*_33_. In this work, fixed equi-biaxial prestretch in the *x*_1_ and *x*_2_ directions is employed; that is, *λ*_1_ = *λ*_2_ = *λ,* and *λ* = 1 is adopted in this work. The unit cell is manifested by the red dashed rectangle, which is composed of material phases *A* and *B* sharing equal volume. The initial length of the unit cell *H = H*^(*A*)^ *+ H*^(*B*)^.

As the above formulations state, the geometrical and material characteristics can be tuned by the applied mechanical load, in which case the controllable dispersion relations of longitudinal waves are induced by manipulating the applied prestress. As shown in Figure 1, the considered compressible elastomers are denoted by material phase *A* and phase *B.* For simplicity, the initial lengths of them denoted by *H*^(*A*)^ and *H*^(*B*)^ are equal, which is assumed to be 0.5 mm. The 1D soft PnC is subject to fixed equi-biaxial prestretch in the *x*_1_ and *x*_2_ directions, indicating that *λ*_1_ = *λ*_2_ = *λ.* Also, *λ* = 1 is adopted in the subsequent project for completing the inverse design. The external prestress is denoted by *σ*_33_, which is designed to range from the negative value to the positive value, corresponding to the compressible and stretching effects.

Taking into account the deformation, the length of each phase is redefined as $h^{\left(p\right)}=H^{\left(p\right)}\lambda_{3}^{\left(p\right)}$ where $\lambda_{3}^{\left(p\right)}$ is the stretch ratio of phase *p* (*p* = material phase *A* or *B*) is in the direction of *x*_3_, and thus the instantaneous lattice constant *h* is obtained by *h* = *h*^(*A*)^ + *h*^(*B*)^. Obviously, in this case, the deformation gradient **F** can be derived as: $F=\mathrm{diag}\left[\lambda ,\;\lambda ,\;\lambda_{3}^{\left(p\right)}\right]$. Based on the expressions of the invariants with the free energy density function and the Jacobian, the prestress *σ*_33_ is able to be formulated as:

(8) $$\sigma_{33}=\mu^{\left(p\right)}[{\left(\lambda_{3}^{\left(p\right)}\right)}^{2}-\lambda^{-4\beta^{\left(p\right)}}{\left(\lambda_{3}^{\left(p\right)}\right)}^{-2\beta^{\left(p\right)}}]/(\lambda^{2}\lambda_{3}^{\left(p\right)})$$

Focusing on the longitudinal wave propagation, Equation (6) is rewritten as:

(9) $$C_{3333}\partial^{2}{\bar{u}}_{3}/\partial x_{3}^{2}=\rho \partial^{2}{\bar{u}}_{3}/\partial t^{2}$$

here, ${\bar{u}}_{3}$ is the longitudinal incremental displacement, and *C*_3333_ is the component of the instantaneous elastic moduli tensor. Employing the expression of **F** and Equation (7), the modulus of material phase *p*-$C_{3333}^{\left(p\right)}$ can be formulated as:

(10) $$C_{3333}^{\left(p\right)}=\mu^{\left(p\right)}[{\left(\lambda_{3}^{\left(p\right)}\right)}^{2}+(2\beta^{\left(p\right)}+1)\lambda^{-4\beta^{\left(p\right)}}{\left(\lambda_{3}^{\left(p\right)}\right)}^{-2\beta^{\left(p\right)}}]/(\lambda^{2}\lambda_{3}^{\left(p\right)})$$

As can be seen, the magnitude of *σ*_33_ determines the magnitude of $\lambda_{3}^{\left(p\right)}$, meanwhile the variation of $\lambda_{3}^{\left(p\right)}$ plays an important role in controlling the equivalent modulus $C_{3333}^{\left(p\right)}$, thereby realizing tuning band gaps of the soft PnCs through controlling *σ*_33_.

#### 2.2.2. The Modelling Based on TMM for Band Gaps

**Figure 2:**
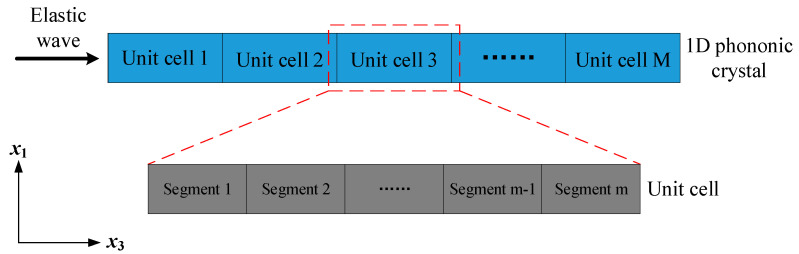
Schematic descriptions of waves propagating through the unit cell. Here, the unit cell is assumed to consist of multiple segments to make a more general statement to formulate the TMM model.

Figure 1 describes the unit cell composed of two segments filled with material phases *A* and *B* for brevity to discover the relation between the external load and mechanical properties. Here, the unit cell is assumed to consist of multiple segments to make a more general statement to formulate the TMM model focusing on longitudinal waves. The unit cell considered in the finite soft PnCs is illustrated in Figure 2.

The elastic wave propagates along the longitudinal direction whose characteristics are governed by the following:

(11) $$C_{3333}\partial^{2}u(x_{3},t)/\partial x_{3}^{2}=\rho \partial^{2}u(x_{3},t)/\partial t^{2}$$

where *u* represents the displacement in the *x*_3_ direction. Assuming harmonic motion, Equation (11) can be rewritten as:

(12) $$C_{3333}\partial^{2}U(x_{3})/\partial x_{3}^{2}=-\rho \omega^{2}U(x_{3})$$

According to Bloch’s theory, the periodic boundary condition requires *U*(*x*_3_ + *h*) = *e^iqh^U*(*x*_3_). Applying this boundary condition to TMM, the eigenvalue equation can be obtained:

(13) $$\left|T-e^{iqh}I\right|=0$$

where *h* is the instantaneous length of the unit cell and *q* is the wavenumber of the Bloch wave. In Equation (13), the matrix **T** denotes the transfer matrix, and the detailed derivations can be found in Appendix A. The real part and imaginary part of *q* are obtained by solving Equation (13), which regards *q* as the eigenvalue for given frequencies *f*. In this case, the location, width, and attenuation level of wave band gaps can be examined. In order to validate the proposed TMM model, the material properties are employed from Ref. [88], and the normalized frequency Ω is adopted to eliminate the effects of the unit cell length on the band structure. Also, for brevity's purpose, the prestress is normalized by *σ*_0_ = *σ*_33_/*μ_A_* and exerted at the two ends of the unit cell. These assumptions remain still unless otherwise specified in the following. Appendix B provides more details about validating the proposed TMM model.

## 3. Implementation of the Inverse Design Based on DL

The inverse design of the present work is expected to provide multiple feasible topological layouts with different prestresses that can share the same prescribed band gaps. The band gap properties are characterized by the bounding frequencies of interested stop bands in an automatic manner. It is noted that both the topology and the prestress being responsible for controlling wave propagation are required as the outputs here, showing more flexibility and complexity in contrast to the studies using the traditional TO conducted under constant external load. The inputs are assigned as the lower and upper bounding frequencies, and in this work, they are classified into three cases: the lower and upper boundary of the first stop band (Ω_1*l*_, Ω_1*u*_), that of the second stop band (Ω_2*l*_, Ω_2*u*_), and the combination of them (Ω_1*l*_, Ω_1*u*_, Ω_2*l*_, Ω_2*u*_). The DL-based models that correspond to the above-specified conditions are trained independently for outputting the topology layout and the prestress.

### 3.1. Description and Acquisition of the Dataset

**Figure 3:**
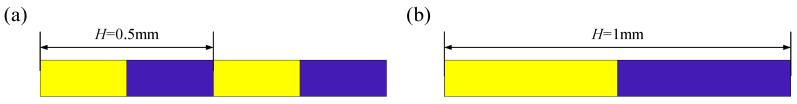
The 1D PnC whose initial unit cell length *H* is assumed as (**a**) 0.5 mm and (**b**) 1 mm, respectively. The analysis of band gaps based on such configurations is conducted to explain the reason for adopting *N* = 97 (where the color blue represents the element filled with phase *A* and yellow represents phase *B*).

In order to facilitate investigating the effects of topological distribution on the band gaps of soft PnCs, the unit cell is assumed to be composed of two soft compressible materials, *A* and *B*, as described before and discretized into *N* elements that share equal length of *H*/*N*. *H* is designed as 1 mm, denoting the unit cell length at the undeformed state. In order to avoid the emergency of topological layouts manifesting that the unit cell consists of multiple representative units, the number of divisions *N* is taken as a prime number. For example, as shown in Figure 3a, two repeatable elements constitute the unit cell, and thus the actual unit cell length accounts for 0.5 of *H* in such a displayed configuration. Hence, in this case, the absolute frequencies calculated by TMM are twice as large as those derived from the soft PnC shown in Figure 3b. However, the unit cell length *H* participating in normalizing the bounding frequencies is kept still during the whole procedure of training DL models, which causes incorrect inputs to the inverse network. Thus, in the present work, *N* is assumed to be 97, that is, the unit cell of the soft PnC with applied prestress is divided into 97 linear elements.

**Figure 4:**
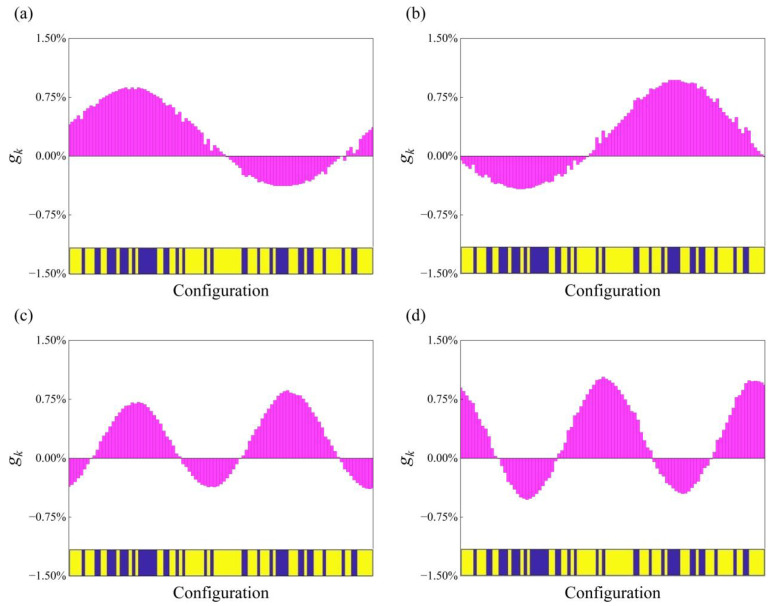
The sensitivity information of the bounding frequencies: (**a**) Ω1l, (**b**) Ω1u, (**c**) Ω2l, and (**d**) Ω2u for a randomly selected material distribution. The configuration is displayed close to the bottom of each plot, and the amplitude of each slender column indicates the sensitivity to the corresponding division (where the color blue represents the element filled with phase A and yellow represents phase B).

Assuming that *a_k_* (*k* = 1–97) represents the phase of filling material. The division is filled by phase *A* via setting *a_k_* = 0 and phase *B* via setting *a_k_* = 1. It is evidently seen that 2^97^ distributions can be achieved without considering prestress, exhibiting higher dimensional mapping complexity of the inverse problem. To depict the effects of topology layouts more intuitively, a material distribution is randomly selected, and the sensitivity information to each divided element is derived to indicate the robustness of the interested inverse problem. As the material distribution is described by a set of discrete variables *a_k_* (*k* = 1–97) whose values can only be 0 or 1, the sensitivity of the bounding frequencies **Ω** = (Ω_1*l*_, Ω_1*u*_, Ω_2*l*_, Ω_2*u*_) with respect to *a_k_* is calculated by the following equation:

(14) $$g_{k}=\left(\Omega^{\prime }-\Omega \right)./\left(\left(a_{k}^{\prime }-a_{k}\right)\Omega \right)\times 100\%$$

where $a_{k}^{\prime }$ represents the reverse of *a_k_*, that is, if *a_k_* = 0 then $a_{k}^{\prime }=1$ and vice versa. $\Omega^{\prime }$ represents the corresponding bounding frequencies calculated based on the material distribution where only *a_k_* is replaced by $a_{k}^{\prime }.$ And for offering a fair evaluation, the relative difference is applied as formulated in Equation (14). The results of sensitivity information are shown in Figure 4. It can be seen that the sensitivity to each element is different, and the maximum is about 1%, which also reflects the complexity of implementing the inverse design to generate the topology layouts for on-demand band gaps.

Apart from the material distribution, the normalized prestress *σ*_0_ increasing from −1 to 1 is sampled with an interval of 0.1. The discrete variables determining the topological layout are associated with the continuous variable reflecting external load together as the output vector to the inverse design. In total, the amount of 63,000 data samples where the material distributions are randomly generated is taken into account, and the corresponding dispersion relations are calculated by the established TMM model to compose the entire dataset, 70% of which is selected as the training dataset. The remaining 30% is divided into two parts, equally, that are used as the validation dataset and test dataset, respectively.

### 3.2. The Framework of the Inverse Design

The CVAE that is generative is employed to learn the mapping from the bounding frequencies of stop bands to the soft PnCs described by the material distribution and applied prestress. To enhance the stability of training the CVAE and further acquire optimal soft PnCs for on-demand requirements, the forward DNN model is trained firstly, and subsequently, the task of filtering out the soft PnCs that do not fall within admissible stop bands as well as ranking the generated candidates according to the wave performance is fulfilled. The DNN based on the MLP architecture is proposed; given the prestress and distribution, the forward model is able to predict the band diagram, functioning as a surrogate model of TMM simulations.

**Figure 5:**
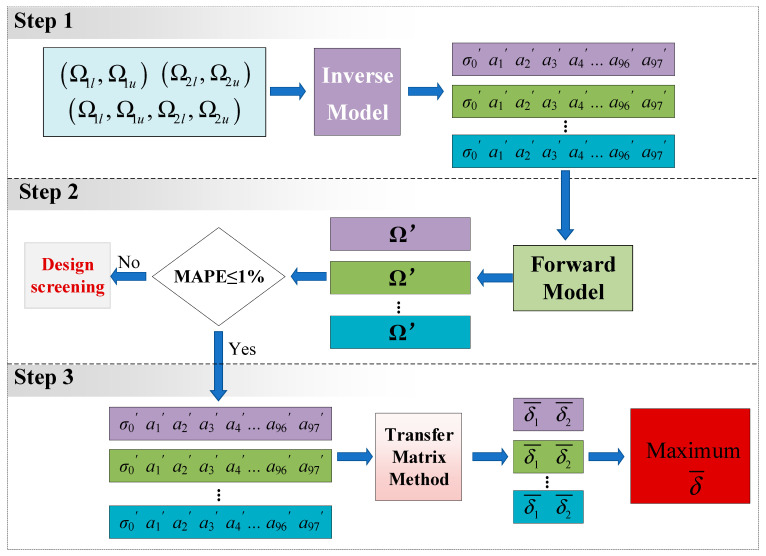
Schematic diagram of the framework of the intelligent inverse design of soft PnCs. The first step illustrates the basic function of the inverse model, that is, outputting different designs to the same input, while the second step and the third step are conducted after validating the accuracy of the inverse model, which is responsible for achieving on-demand goals of tunable band gaps by utilizing the generalized performance of the inverse model.

Employing the well-trained forward DNN and CVAE concurrently, the proposed DL-based inverse design framework is visualized in Figure 5, consisting of three steps to accelerate the exploration of on-demand soft PnCs. Regarding the band gaps prescribed by special requirements, the established CVAE at the first step allows the generation of multiple classes of topological layouts with predicted prestress. It is worth noting that the target characteristics of band gaps can be specified by researchers, and the training of the CVAE is performed three times independently when the input vector is designed as (Ω_1*l*_, Ω_1*u*_), (Ω_2*l*_, Ω_2*u*_), and (Ω_1*l*_, Ω_1*u*_, Ω_2*l*_, Ω_2*u*_), respectively. The second step is responsible for filtering out the unsatisfactory soft PnCs among the massive generated candidates with the aid of forward DNN, and in this work, the well-trained inverse model has been performed at a maximum of 5000 times. The error limit of MAPE less than 1% is adopted, and the soft PnCs whose bounding frequencies exceed this criterion will be removed. The expression of MAPE can be found in Equation (15). As can be seen, the inverse design with respect to special requirements of real band diagrams can be solved by accomplishing step 1 and step 2. Nevertheless, the real part of dispersion relations is unable to estimate the attenuation level of elastic waves inside the stop bands. Thus, step 3 is added to the framework, which aims at undertaking the task of ranking the feasible unit cells in terms of wave attenuation performance. The TMM is employed again at this step to calculate the attenuation constant *δ*, which determines the decaying degree of Bloch waves for given frequencies. Based on the comparison results of *δ* among the generated feasible PnCs, the topological layout within applied prestress that can possess the maximum attenuation constant is emerging and regarded as the desired solution exhibiting enhanced performance.

(15) $$\mathrm{MAPE}=\frac{1}{n}\left(\left|\left(\Omega^{\prime }-\Omega \right)./\Omega \right|\right)\times 100\%,\;n-\mathrm{thedimension}\;\mathrm{of}\;\mathrm{predicted}\;\mathrm{frequencies}$$

### 3.3. The Architecture of Forward and Inverse Models

Focusing on the forward path, the DL-based model is more likely to be established in a deterministic supervised learning way to predict the nonlinear dynamic behavior of various configurations of soft PnCs with a reasonable trade-off in accuracy. Therefore, the forward model implemented follows the MLP architecture and consists of one input layer, five fully connected layers attached with activation functions (HardSwish), and one output layer. The hidden layers contain 196, 196, 196, 196, and 160 neurons, respectively, and the input is represented by the prestress combined with material distribution, while the output is the bounding frequencies of the first and second stop bands. The optimization algorithm is used with a learning rate of 0.0005 to adjust the hyperparameters automatically to minimize the mean absolute error (MAE) loss function that is formulated as follows:

(16) $$L_{\mathrm{DNN}}=\frac{1}{4m}\sum_{i=1}^{m}\left(\left|\Omega_{1l}-\Omega_{1l}^{\prime }\right|+\left|\Omega_{1u}-\Omega_{1u}^{\prime }\right|+\left|\Omega_{2u}-\Omega_{2u}^{\prime }\right|+\left|\Omega_{2l}-\Omega_{2l}^{\prime }\right|\right)$$

where *m* denotes the number of samples in the dataset.

In order to ensure the generation of multiple feasible candidates for the same input of given bounding frequencies, the DL-based model in the inverse path is used in the form of the CVAE consisting of the encoder and decoder parts. Under the behavior condition of target bounding frequencies **Ω**, the components of design variables, including the continuous prestress exerted at the unit cell and the discrete variables determining the topological layout, are fed into the encoder for mapping them to the latent variables ***z*** following the Gaussian distribution characterized by the expectancy ***α*** and standard deviation ***γ***. Then the latent variables ***z*** are concatenated with targets **Ω**, which is regarded as the input vector to the decoder for generating soft PnCs in terms of the topology with prestress. The viable and diverse capacities are induced by minimizing the reconstruction error and KL divergence. Reconstruction error *L*_CVAE1_ measures the difference between the reconstructed and the original samples, while KL divergence is used to evaluate the difference between the distribution of latent variables ***z*** and the standard normal distribution. By optimizing the error terms, the CVAE can learn the mapping between the band gaps and topological layouts with applied prestress. It is worth noting that the Sigmoid function is displayed at the end of the decoder as the discrete nature of the variables representing the topological layout needs to be restored. Furthermore, the well-trained forward model is in tandem with the CVAE, which is responsible for predicting the bounding frequencies of the generated soft PnCs.

For brevity purposes, the architectures of both the forward and inverse models are illustrated in Appendix C, from which it can be seen that the error *L*_CVAE2_ between the predictions by the forward model and targets also takes part in calculating the loss function when training the inverse model. Therefore, the loss function *L*_total_ of the entire inverse design contains error terms along both the inverse and forward paths, in which case *L*_total_ can be formulated as:

(17) $$L_{\mathrm{total}}=L_{\mathrm{CVAE}1}+\chi \left(\mathrm{KL}+\sum_{i=1}^{m}\sum_{k=1}^{n}\left(1/\gamma_{ik}^{2}\right)\right)+0.5L_{\mathrm{CVAE}2}$$

where:

(18) $$L_{\mathrm{CVAE}1}=\sum_{i=1}^{m}\left|\sigma_{0i}-\sigma_{0i}^{\prime }\right|+\sum_{i=1}^{m}\sum_{j=1}^{N}\left|a_{ij}-a_{ij}^{\prime }\right|,\;\mathrm{KL}=\left[-\sum_{i=1}^{m}\sum_{k=1}^{n}\left(1+\log\left(\gamma_{ik}^{2}\right)-\gamma_{ik}^{2}-\alpha_{ik}^{2}\right)\right]/(2mn)$$

(19) $$L_{\mathrm{CVAE}2}=\left\{\begin{array}{l} \left|\Omega_{1l}-\Omega_{1l}^{\prime }\right|+\left|\Omega_{1u}-\Omega_{1u}^{\prime }\right|,\;\;\;\mathrm{Target}:1^{\mathrm{st}}\;\mathrm{BG}\;\\ \left|\Omega_{2l}-\Omega_{2l}^{\prime }\right|+\left|\Omega_{2u}-\Omega_{2u}^{\prime }\right|,\;\;\;\mathrm{Target}:2^{\mathrm{nd}}\;\mathrm{BG}\\ \left|\Omega_{1l}-\Omega_{1l}^{\prime }\right|+\left|\Omega_{1u}-\Omega_{1u}^{\prime }\right|+\left|\Omega_{2l}-\Omega_{2l}^{\prime }\right|+\left|\Omega_{2u}-\Omega_{2u}^{\prime }\right|,\;\mathrm{Target}:1^{\mathrm{st}}\;\mathrm{and}\;2^{\mathrm{nd}}\;\mathrm{BGs} \end{array}\right.$$

In the above equations, *m* and *n* represent the number of samples in the training dataset and the number of elements in the variable vector ***z***, respectively. The weight of KL divergence *χ* is the hyperparameter, and its value is determined via a search process on the validation set. In detail, the optimal value is explored within the specified search spaces using Bayesian single-objective optimization from Optuna, with a total of 200 trials: *χ* = (0.001, 0.1, step = 0.001). The Nondominated Sorting Genetic Algorithm III (NSGA-III) in Optuna is used for sampling, and finally, this hyperparameter yields the best performance at *χ* = 0.06. Additionally, during the optimizing process, each trial takes 100 epochs. Regarding the other hyperparameters of the networks, they are determined using heuristic methods.

After assembling the architectures of the forward and inverse models, they are trained in sequence and terminated at 1500 epochs and 1000 epochs, respectively. The accuracy of DL-based models is then examined, and the details are given in Appendix C, from which the superior capacity to generate feasible designs is observed by combining the forward DNN and inverse CVAE. Moreover, Table 1 provides the cost time for training models as well as predicting bounding frequencies or generating soft PnCs. As can be seen, the proposed models exhibit extremely high computation efficiency.

## 4. Extended Investigation on Topology Design for Soft PnC

The accuracy of the inverse network has been proved already. Moreover, to demonstrate the generalized performance and deeply explore the design potential under specified conditions, the band gaps are customized with “on-demand” requirements and then employed as targets. By using the well-trained models, this section determines to motivate the generation of multiple innovative candidates of soft PnCs corresponding to these assigned targets.

### 4.1. Inverse Design for Artificial Band Gaps

In this subsection, three cases termed Case I, Case II, and Case III are taken into account, where the generated unit cells are expected to possess prescribed bounding frequencies of the first stop band, the second stop band, and both simultaneously. To verify the generalized performance, the boundaries are constructed in a man-made manner. Firstly, an averaging operation is applied to the bounding frequencies of four data samples randomly selected from the dataset. Then the numerical values of operated boundaries are reserved to two decimal places to ensure that the formed band gaps are beyond the entire dataset. Performing the mentioned instructions, nine groups of artificial band gaps can be achieved and regarded as on-demand inputs. As a result, Case I, Case II, and Case III are divided into different categories: Case I–I, Case I–II, Case I–III, Case II–I, Case II–II, Case II–III, Case III–I, Case III–II, and Case III–III. The target for each category can be found in Table 2.

**Figure 6:**
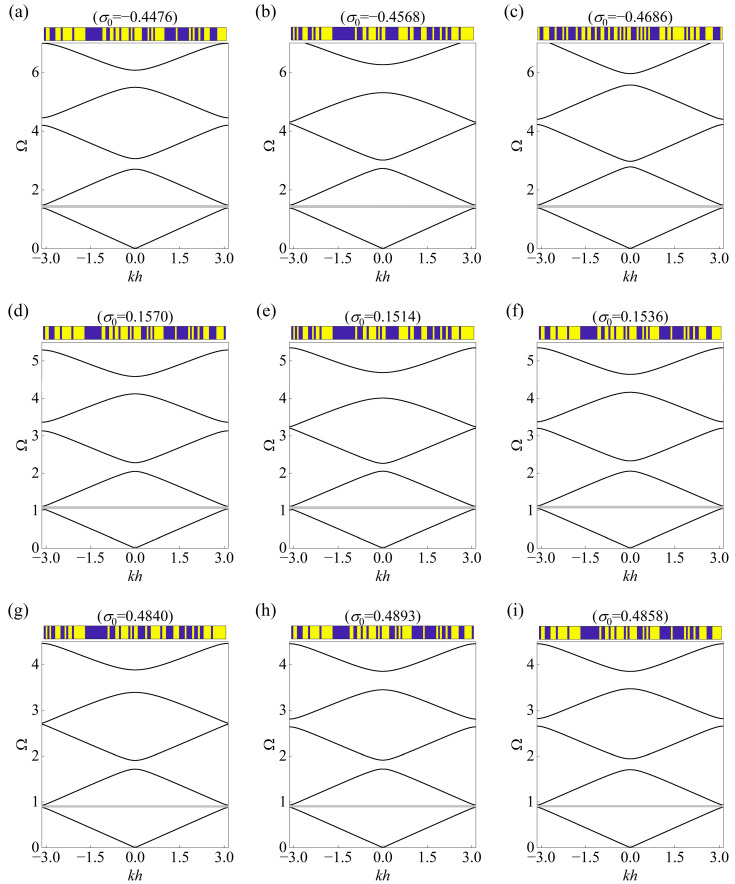
Three solutions of topological layout with prestress for the same input of bounding frequencies specified in Table 2. The continuous curves represent the corresponding real band diagrams in the mentioned cases of (**a**–**c**): Case I–I, (**d**–**f**) Case I–II, and (**g**–**i**) Case I–III. The generated configuration is displayed at the top of each plot, where the color blue represents the element filled with phase *A* and yellow represents phase *B.* Also, the gray regions denote the prescribed stop bands.

**Figure 7:**
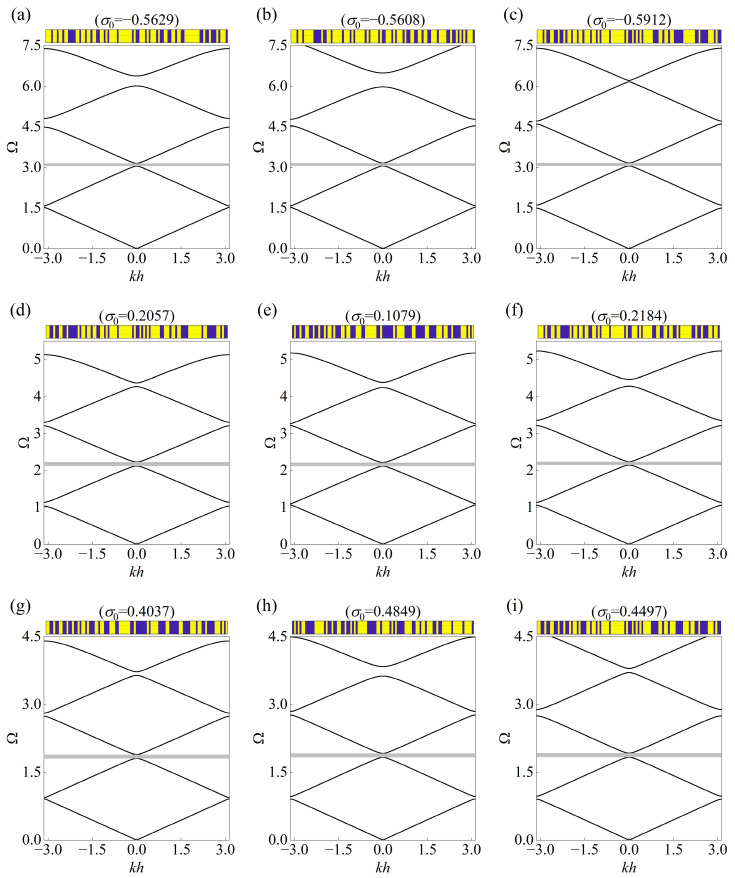
Three solutions of topological layout with prestress for the same input of bounding frequencies specified in Table 2. The continuous curves represent the corresponding real band diagrams in the mentioned cases of (**a**–**c**): Case II–I, (**d**–**f**) Case II–II, and (**g**–**i**) Case II–III. The generated configuration is displayed at the top of each plot, and the gray regions denote the prescribed stop bands.

**Figure 8:**
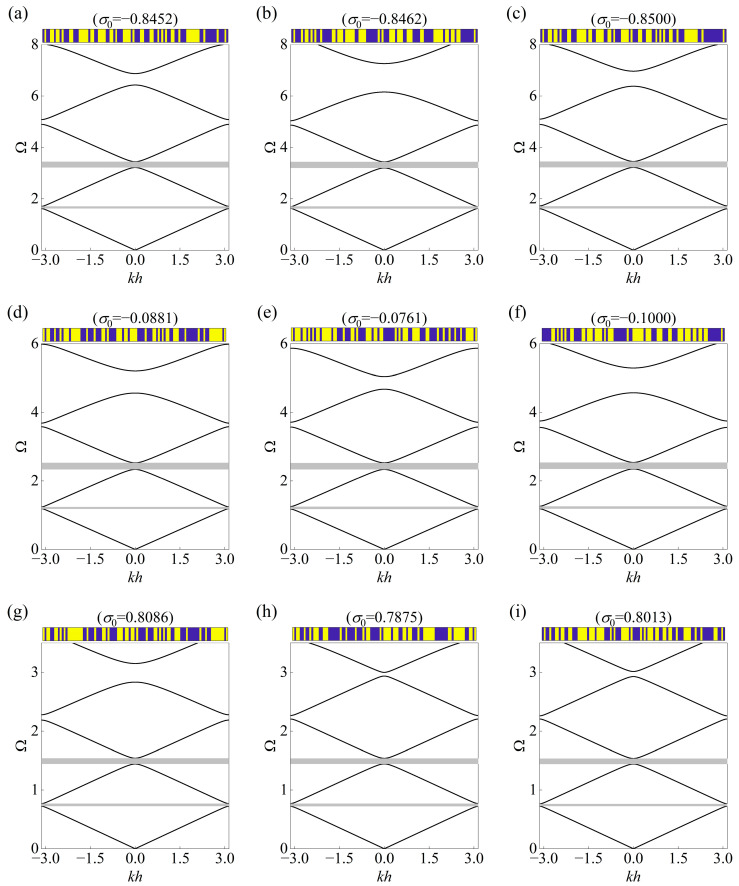
Three solutions of topological layout with prestress for the same input of bounding frequencies specified in Table 2. The continuous curves represent the corresponding real band diagrams in the mentioned cases of (**a**–**c**): Case III–I, (**d**–**f**) Case III–II, and (**g**–**i**) Case III–III. The generated configuration is displayed at the top of each plot, and the gray regions denote the prescribed stop bands.

The inherent probabilistic feature of the CVAE allows multiple generations, and here three types of the topology accompanying prestress are provided as the solutions for brevity to the inverse design problem concerning the same input. The topological layouts of the unit cells and the corresponding band diagrams are presented in Figure 6, Figure 7 and Figure 8, where the stop bands are highlighted by gray regions. As can be seen, the generated unit cells exhibit unique configurations but share overlapping stop bands in accordance with the targets given in Table 2, which demonstrates the diversity and generalized performance of the established framework.

In addition, it can be noticed that the outputted prestress also presents different levels even the same input is assigned, and both compression and stretch effects appear as the prior condition to tune the band diagram among the total 27 groups. Surprisingly, the fourth stop band, located between the fourth and fifth dispersion curves, as found from the plots, has become inconsistent with each other. For example, the fourth stop band in Figure 6a starts at 5.49164 and ends at 6.07446, but the location of that in Figure 6b ranges from 5.31006 to 6.26236. This can be explained by the differences between the topological layouts, which comply with the mechanism of Bragg scattering and indicate the complexity of the considered inverse problem.

### 4.2. Inverse Design for Broadening and Lowering Band Gaps

By taking advantage of the diversity ability of the present network, the topology with applied prestress that can exhibit lower and wider stop bands is expected to be generated. To deeply explore the potential for providing innovative structures, the bounding frequencies reflecting the location and bandwidth of the demand-satisfying band gaps are achieved by a step-by-step approach. Specifically, initial stop bands are randomly selected out of the entire dataset at first, and then the upper boundary remains still while the lower boundary is continuously reduced by a gradual deduction. As a result, the bandwidth is broadened, and the central position of the stop bands is moving towards lower frequencies. The newly formed band gap in each step is regarded as the input, in which case the well-trained CVAE is required to provide multiple feasible candidates. The accuracy is assured with the aid of the forward network through filtering out the designs that violate the criterion defined by MAPE defined by Equation (15) of being less than 1%. This manipulation for decreasing the lower bounding is terminated until the values of MAPE of all generated structures are larger than 1%. Finally, the inverse design is completed with high accuracy and efficiency in regard to searching for soft PnCs corresponding to lower and wider band gaps. Three cases named Case ①, Case ②, and Case ③ are investigated, respectively, which corresponds to the condition that the above operations are performed on the first and second stop bands simultaneously, the first stop band only, and the second stop band only.

As shown in Table 3, the initial ranges of the first and second stop bands in Case ① are 1.75–1.80 and 3.50–3.70, the lower bounding frequencies of which are deducted by 0.05 and 0.1 per step separately. For brevity purposes, one presentation of the topological layout with the outputted prestress is illustrated at each step. It is clearly seen that the first and second stop bands continuously shift to lower frequencies and are broadened until they reach the range covering 1.55–1.80 and 3.10–3.70, to which the CVAE cannot provide material distribution with satisfactory accuracy. The final layout corresponds to the first stop band, ranging from 1.60 to 1.80, and the second stop band, from 3.20 to 3.70, whose absolute bandwidth is 4 times and 2.5 times larger than that of the initial stop band, while the relative bandwidth is merely 4.2 times and 2.6 times larger. Moreover, the stop bands can be further expanded in Case ② and Case ③ when employed as the individual target. It is evident from Table 4 to Table 5 that the absolute bandwidth of the first stop band can approach 5 times larger as the upper boundary is confined to 1.80. Meanwhile, the second stop band can be increased to 3 times larger as the upper boundary yields at 3.70.

**Figure 9:**
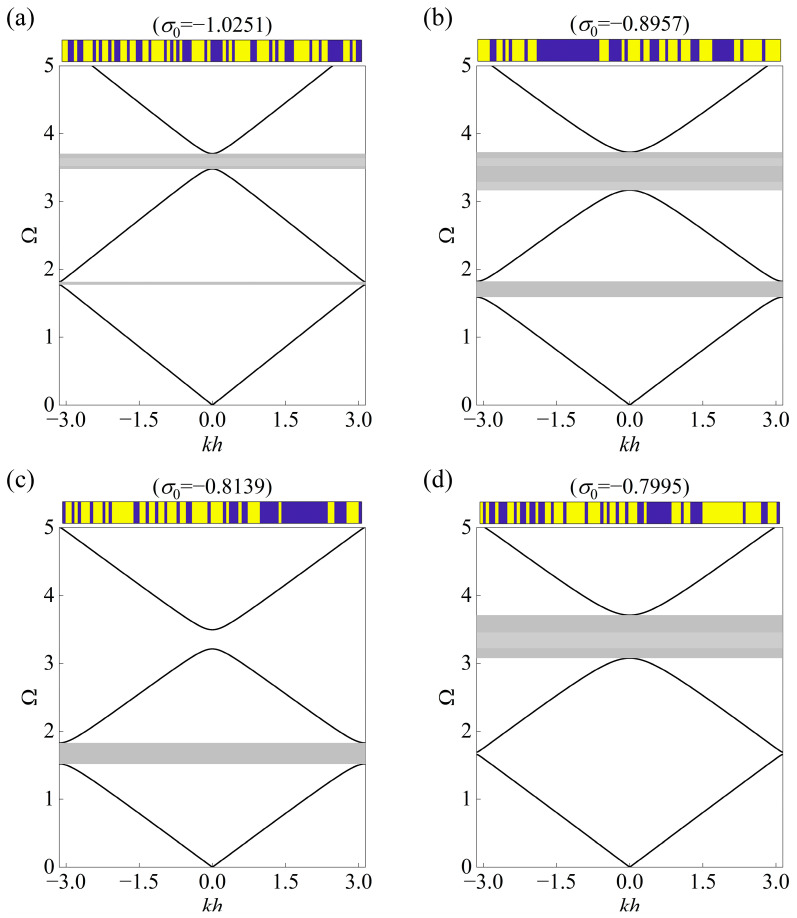
The comparison results between the band diagrams of the (**a**) initial design and offered design at the last step in (**b**) Case ①, (**c**) Case ②, and (**d**) Case ③, where the gray regions denote the stop bands of interested order. The initial and generated configurations with prestress are displayed at the top of each plot.

In addition, the predicted compression stress is found to be gradually decreased to lower the band diagram. Figure 9 presents the band structures of the initial design and final designs. From the comparison results, it can be observed that various “on-demand” requirements on the tunable band gaps are realized by the proposed network.

### 4.3. Inverse Design for Enhanced Wave Attenuation

The results of the real band structure shown in Section 4.1 manifest that the unit cells sharing merely the same first and second stop bands can still present distinctive features of dispersion curves, which further suggests the attenuation performance inside the considered stop bands is not completely consistent with each other. To demonstrate this deduction, the attenuation performance of the generated structures in Case I–I, Case I–II, and Case I–III is evaluated by the attenuation constant *δ*, respectively, via using the TMM model again. As a result, the attenuation peaks of the first stop band are 0.0844, 0.0609, and 0.1056, and those of the second stop band are 0.1355, 0.2262, and 0.2085. Obviously, the bigger the value of *δ*, the more intensive the degree of prohibiting wave propagation can be achieved. This evident difference between various feasible designs within identical bounding frequencies makes it possible to find the optimal topological layout with improved wave attenuation performance.

The generated unit cells are explored by comparing the average attenuation constant $\bar{\delta }$ over the target stop bands. The candidate possessing the maximization of $\bar{\delta }$ is regarded as the desired solution in this subsection. In order to add the flexibility of this inverse problem, the average attenuation constant is formulated as:

(20) $$\bar{\delta }=n_{1}{\bar{\delta }}_{1}+n_{2}{\bar{\delta }}_{2}$$

where ${\bar{\delta }}_{1}$ and ${\bar{\delta }}_{2}$ represent the mean value of the attenuation constant over the first-stop band and second-stop band, respectively.

Based on Equation (20), three conditions are taken into account that correspond to (*n*_1_, *n*_2_) = (1, 0), (*n*_1_, *n*_2_) = (0, 1), and (*n*_1_, *n*_2_) = (0.5, 0.5). The final design, characterized by the topology layout and applied prestress, is expected to be found under each condition. Here, the target bounding frequencies are derived from Table 2; as stated before, these are randomly selected beyond the training, validation, and test datasets. Now, the considered three conditions are termed Case I-(i), (ii), (iii), Case II-(i), (ii), (iii), and Case III-(i), (ii), (iii). In detail, Cases I, II, and III correspond to the bounding frequencies of the first and second stop bands that are (1.62, 1.71, 3.24, 3.41), (1.18, 1.26, 2.35, 2.54), and (0.72, 0.76, 1.44, 1.55), while the sequence numbers (i), (ii), and (iii) denote the selection of the parameter group of (*n*_1_, *n*_2_) described before.

**Figure 10:**
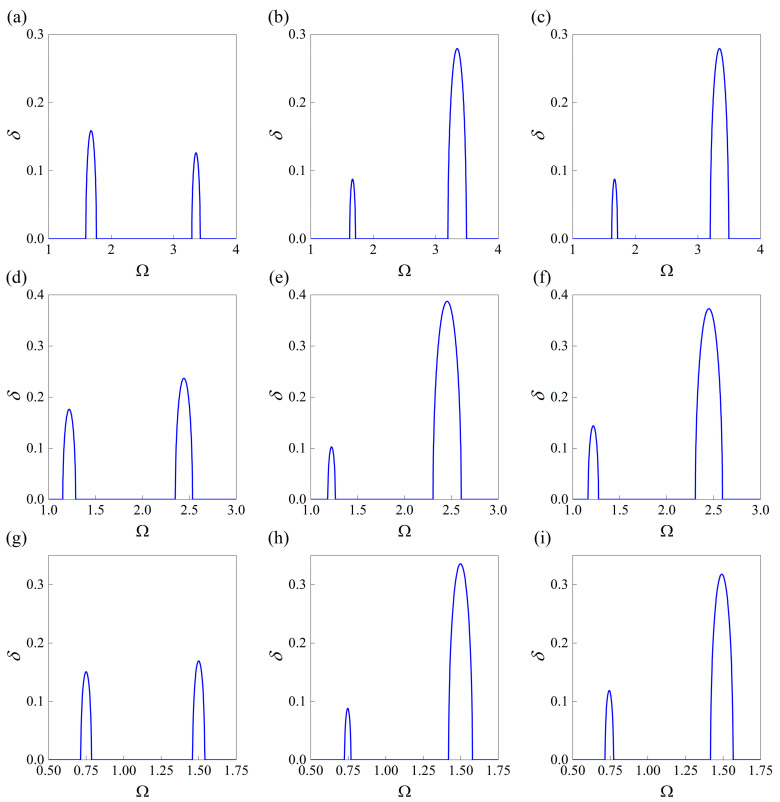
The attenuation diagram for given frequencies, computed based on the configuration and prestress provided from Table 7 to Table 9, whose target is assigned according to (**a**) Case I-(i), (**b**) Case I-(ii), (**c**) Case I-(iii), (**d**) Case II-(i), (**e**) Case II-(ii), (**f**) Case II-(iii), (**g**) Case III-(i), (**h**) Case III-(ii), and (**i**) Case III-(iii).

After running the well-trained inverse model 5000 times in each case, the generated structures that do not satisfy the error limit of being less than 1% of MAPE are filtered out to ensure accuracy. After filtering, there are 4515, 4641, and 3756 feasible designs to be retained in Case I, Case II, and Case III, respectively. By comparing the target values defined in Equation (20) among the feasible designs, the corresponding maximums and minimums of the average attenuation constants are obtained and given in Table 6. As can be seen, whether focusing on the first and second stop bands simultaneously or solely on one stop band, the ability to forbid wave propagation can be significantly improved by searching for the optimal design with a bigger attenuation constant. Correspondingly, the topological layouts with prestress possessing the best attenuation performance among the feasible candidates are given in Table 7, Table 8 and Table 9. Different weight coefficients of (*n*_1_, *n*_2_) can result in diverse optimal designs. To gain a more straightforward understanding of the improved ability, Figure 10 presents the attenuation diagrams of the unit cells given in these tables. It is noted that the presented design in this subsection does not represent the “global optimal” or “local optimal” through searching for the design space using optimization algorithms. It is achieved by making full use of the potential of the inverse network, which is able to find a satisfactory solution at an incomparable speed.

**Figure 11:**
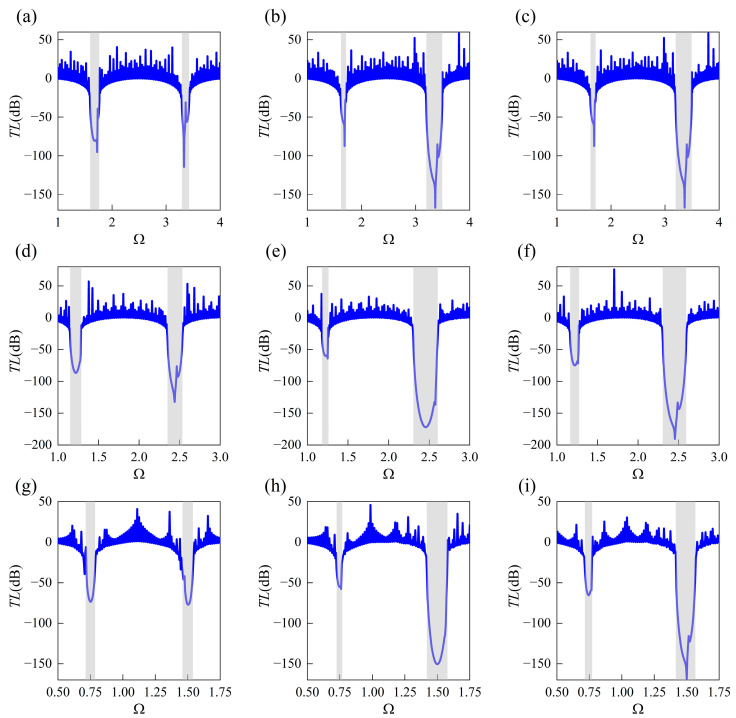
The vibration transmittance of the finite soft PnCs composed of 50 unit cells and applied with CC at the two ends, where blue solid curves denote the transmission loss and gray regions denote the stop bands calculated by the TMM. The corresponding unit cells are employed from the results given in Table 7, Table 8 and Table 9, within the objective of improving wave attenuation performance under different cases: (**a**–**c**): Case I-(i) to Case I-(iii), (**d**–**f**): Case II-(i) to Case II-(iii), and (**g**–**i**): Case III-(i) to Case III-(iii).

Further, to explore the technical value of the design solutions, finite soft structures based on the results of Table 7, Table 8 and Table 9 are taken into consideration to evaluate the vibration insulation performance. Each is constituted by 50 corresponding unit cells with predicted prestress, and the clamped condition (CC) is employed. The harmonic force with unit amplitude displacement is imposed on the left edge, and the vibration isolation is examined by the transmittance *TL* that is expressed in terms of the displacement ratio: *TL* = 20log_10_(*u_r_*/*u_l_*). Here, *u_r_* and *u_l_* represent the displacement at the left side and right side, respectively. The displacement field is acquired by the FEM model. The results of transmittance are shown in Figure 11, where the gray regions denote the stop bands calculated by the TMM. From Figure 11, one can easily observe the location and bandwidth of the attenuation bands with significant insulation are in accord with the stop bands. In addition, as comparing the plots displayed in the same row, the average level for reducing vibration of finite soft PnCs is also found to conform to the objectives assigned in Cases (i), (ii), and (iii), that is, the emphasis is assigned on improving wave attenuation inside the first stop band only, the second stop band only, and the first two stop bands simultaneously, which demonstrates the potential of the proposed framework applied in practical engineering.

## 5. Conclusions

In this work, an innovative design method based on the DL is presented to realize two purposes for designing the soft compressible PnCs: one is for outputting the combination of discrete and continuous design variables in terms of the topological layout and external mechanical prestress, and the other is for automatically offering multiple design solutions with respect to the given target of longitudinal wave band gaps at an extremely fast speed. Based on the nonlinear elastic theory and Bloch-Floquet theory, the TMM model is established to extract the complex dispersion relations of the compressible unit cells. In order to assure the accuracy and diversity performance of DL-based models, the inverse framework is composed of CVAE in tandem with pretrained MLP, where the former, displayed in the inverse path, is responsible for generating candidates and the latter, fixed at the forward path, is regarded as the surrogate model for enhancing the stability and filtering out unfeasible designs.

The average value of MAPE is 0.81% over the test dataset, verifying the accuracy of the inverse network. When the target bounding frequencies are randomly selected beyond the entire dataset, the well-trained CAVE can still output various design schemes characterized by different distributions of material phases applied with prestress, which also validates the superior generalized performance. Furthermore, by making use of the outstanding performance in exploiting design space of the proposed method, this work aims to offer topologies with longitudinal prestress that correspond to lower and broader stop bands. In particular, the generated soft, compressible unit cells possess the first stop band and the second stop band, whose widths are at least 2.5 times larger than that of initial designs. In addition, the presented network shows the capacity to efficiently acquire the combination of the topological layout and external prestress that can induce improved attenuation performance. At first, numerous unit cells are generated by the proposed inverse model. Then they are filtered out according to the accuracy criterion. Eventually, the desired solution is obtained via the ranking in terms of the attenuation constant among the feasible generated candidates. Moreover, the attenuation level is also validated by the vibration insulation of finite soft PnCs. Overall, the proposed method can serve as a potent tool for realizing on-demand soft PnCs, enabling researchers to intelligently control vibration and sound characteristics.

## Figures and Tables

**Table 1:** The cost time for training and performing DL-based models.

	Forward Model		Inverse Model	
	Training	Predicting (9549 data/single data)	Training	Generating (9549 data/single data)
Time (s)	8771.80	8.10/0.00084	8778.12	33.30/0.0035

**Table 2:** The boundaries of stop bands that are regarded as on-demand inputs.

Classifications	The Normalized Bounding Frequencies		
	Case I	Case II	Case III
I	1.39~1.49	3.02~3.15	(1.62~1.71, 3.24~3.41)
II	1.05~1.12	2.12~2.25	(1.18~1.26, 2.35~2.54)
III	0.88~0.93	1.83~1.92	(0.72~0.76, 1.44~1.55)

**Table 3:** The topological layout accompanying prestress at each step for lowering and broadening the target stop bands in Case ①.

	Case ①: (1.75~1.80, 3.50~3.70)	The Output Design Scheme
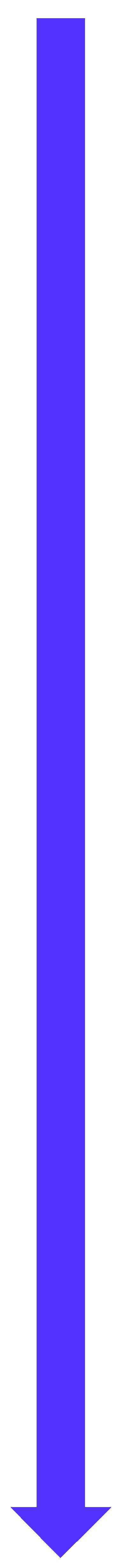	(1.70~1.80, 3.40~3.70)	(*σ*_0_ = −1.0521) 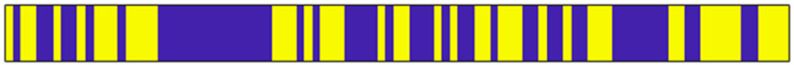
	(1.65~1.80, 3.30~3.70)	(*σ*_0_ = −0.9405) 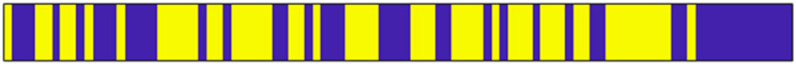
	(1.60~1.80, 3.20~3.70)	(*σ*_0_ = −0.8957) 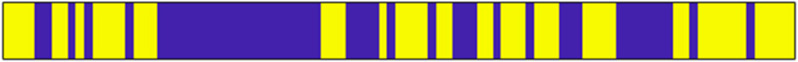
	(1.55~1.80, 3.10~3.70)	NaN

**Table 4:** The topological layout accompanying prestress at each step for lowering and broadening the target stop bands in Case ②.

	Case ②: 1.75~1.80	The Output Design Scheme
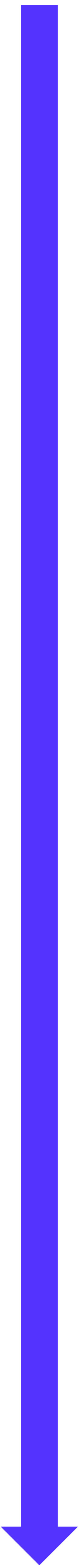	1.70~1.80	(*σ*_0_ = −0.9768) 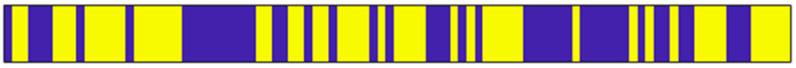
	1.65~1.80	(*σ*_0_ = −0.9228) 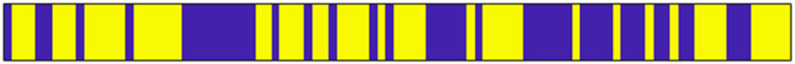
	1.60~1.80	(*σ*_0_ = −0.8689) 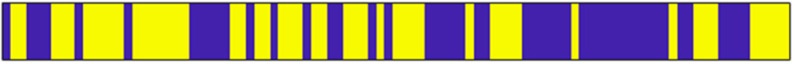
	1.55~1.80	(*σ*_0_ = −0.8139) 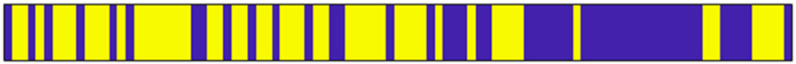
	1.50~1.80	NaN

**Table 5:** The topological layout accompanying prestress at each step for lowering and broadening the target stop bands in Case ③.

	Case ③: 3.50~3.70	The Output Design Scheme
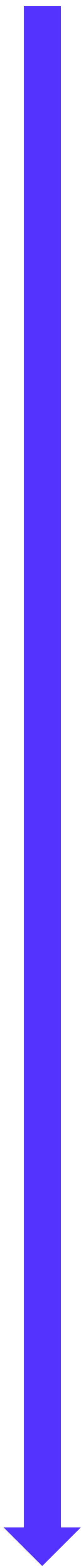	3.40~3.70	(*σ*_0_ = −0.9395) 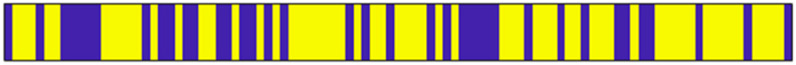
	3.30~3.70	(*σ*_0_ = −0.8889) 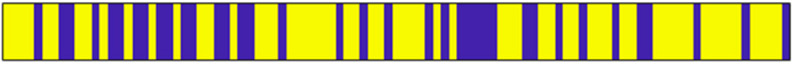
	3.20~3.70	(*σ*_0_ = −0.8453) 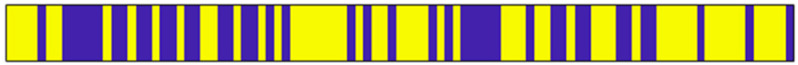
	3.10~3.70	(*σ*_0_ = −0.7995) 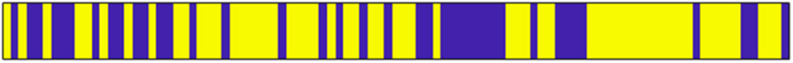
	3.00~3.70	NaN

**Table 6:** The best and worst attenuation performance over target stop bands among generated feasible designs.

	The Average Attenuation Constant Estimating Attenuation Level					
	Maximum in Case I	Minimum in Case I	Maximum in Case II	Minimum in Case II	Maximum in Case III	Minimum in Case III
Case i: (*n*_1_, *n*_2_) = (1, 0)	${\bar{\delta }}_{1}=0.1244$	${\bar{\delta }}_{1}=0.0193$	${\bar{\delta }}_{1}=0.1388$	${\bar{\delta }}_{1}=0.0261$	${\bar{\delta }}_{1}=0.1184$	${\bar{\delta }}_{1}=0.0186$
Case ii: (*n*_1_, *n*_2_) = (0, 1)	${\bar{\delta }}_{2}=0.2188$	${\bar{\delta }}_{2}=0.0296$	${\bar{\delta }}_{2}=0.3050$	${\bar{\delta }}_{2}=0.0963$	${\bar{\delta }}_{2}=0.2656$	${\bar{\delta }}_{2}=0.0891$
Case iii: (*n*_1_, *n*_2_) = (0.5, 0.5)	$\bar{\delta }=0.1441$	$\bar{\delta }=0.0467$	$\bar{\delta }=0.2041$	$\bar{\delta }=0.0816$	$\bar{\delta }=0.1718$	$\bar{\delta }=0.0745$

**Table 7:** The generated unit cells with applied prestress that can produce enhanced attenuation performance over the target band gaps: 1.62–1.71 and 3.24–3.41.

	Design for Improved Wave Attenuation
Case I-(i)	(*σ*_0_ = −0.8496) 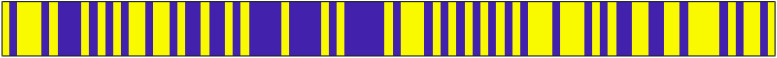
Case I-(ii)	(*σ*_0_ = −0.8413) 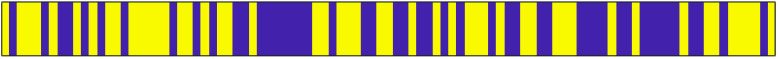
Case I-(iii)	(*σ*_0_ = −0.8413) 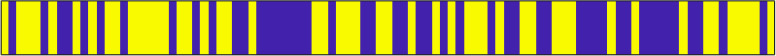

**Table 8:** The generated unit cells with applied prestress that can produce enhanced attenuation performance over the target band gaps: 1.18–1.26 and 2.35–2.54.

	Design for Improved Wave Attenuation
Case II-(i)	(*σ*_0_ = −0.0936) 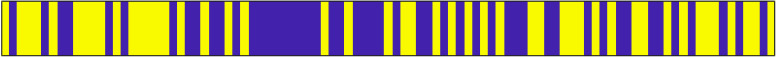
Case II-(ii)	(*σ*_0_ = −0.1137) 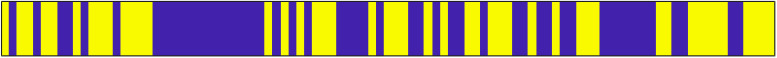
Case II-(iii)	(*σ*_0_ = −0.0892) 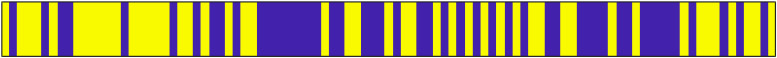

**Table 9:** The generated unit cells with applied prestress that can produce enhanced attenuation performance over the target band gaps: 0.72–0.76 and 1.44–1.55.

	Design for Improved Wave Attenuation
Case III-(i)	(*σ*_0_ = 0.8379) 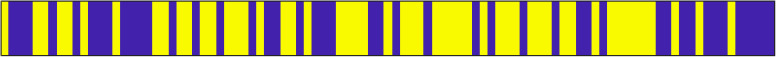
Case III-(ii)	(*σ*_0_ = 0.7719) 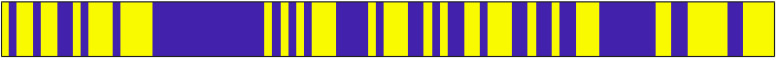
Case III-(iii)	(*σ*_0_ = 0.8142) 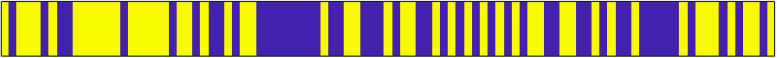

**Figure A1:**
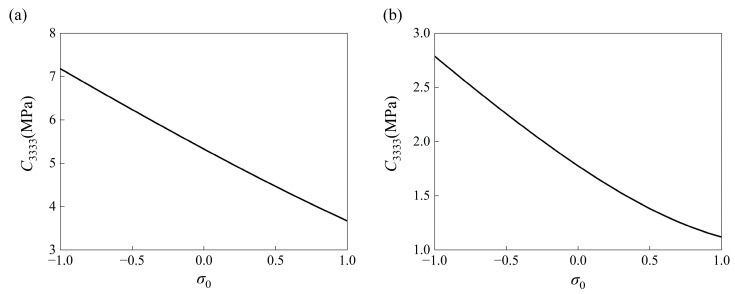
Variations of the simultaneous modulus *C*_3333_ of (**a**) material phase *A* and (**b**) material phase *B* as a function of normalized prestress *σ*_0_ that is increased from −1 to 1.

**Figure A2:**
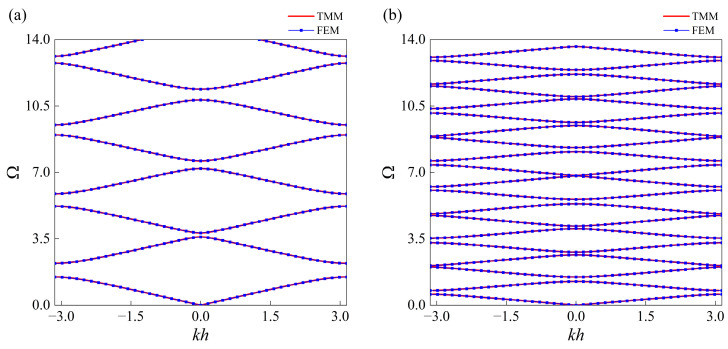
Comparison results of the band structures obtained by TMM and FEM for soft PnCs in the case of (**a**) *σ*_0_= −1 and (**b**) *σ*_0_ = 1, where the dispersion relations from TMM to FEM are represented by the red line and the blue line with symbol, respectively.

**Figure A3:**
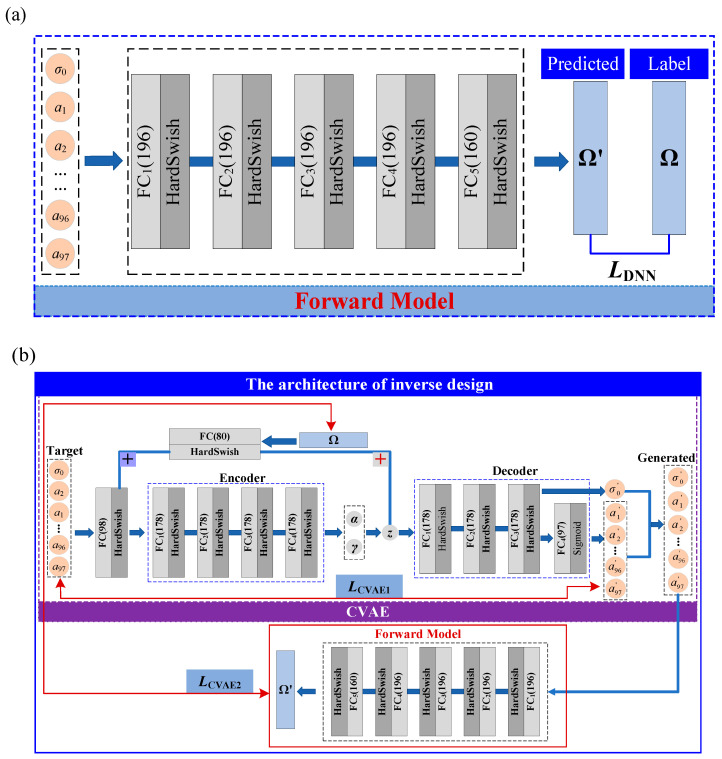
(**a**) The architecture of the forward model is based on the DNN, consisting of one input layer, five fully connected layers attached with activation functions (HardSwish), and one output layer. The hidden layers contain 196, 196, 196, 196, and 160 neurons, respectively. (**b**) The architecture of the inverse model combining CVAE and a well-trained forward DNN. The design variables, including prestress and topological layout, are fed into the encoder for mapping them to the latent variables ***z*** under the behavior condition of target bounding frequencies **Ω**, and then ***z*** is concatenated with targets **Ω**, as inputs fed into the decoder. The well-trained forward model is in tandem with the CVAE; in this case, the loss function during the training of the entire inverse design contains both error terms from inverse and forward models.

**Figure A4:**
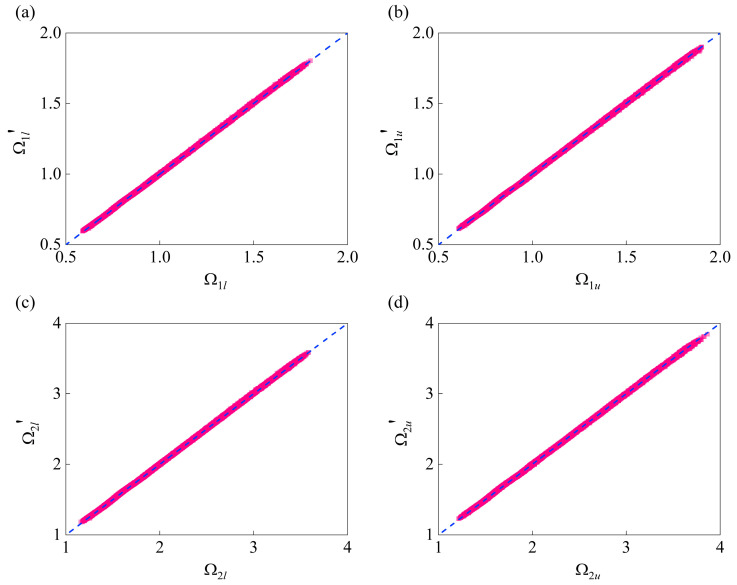
The predictions by the forward DNN and the ground truth of bounding frequencies over all the testing samples. The abscissa in the subfigures denotes the ground truth of bounding frequencies, and the ordinate axis denotes the prediction of bounding frequencies. In detail, (**a**,**b**) are the lower and upper boundaries of the first stop band, and (**c**,**d**) are the lower and upper boundaries of the second stop band. The blue dashed line reflects the physical meaning of “*y* = *x*”.

**Figure A5:**
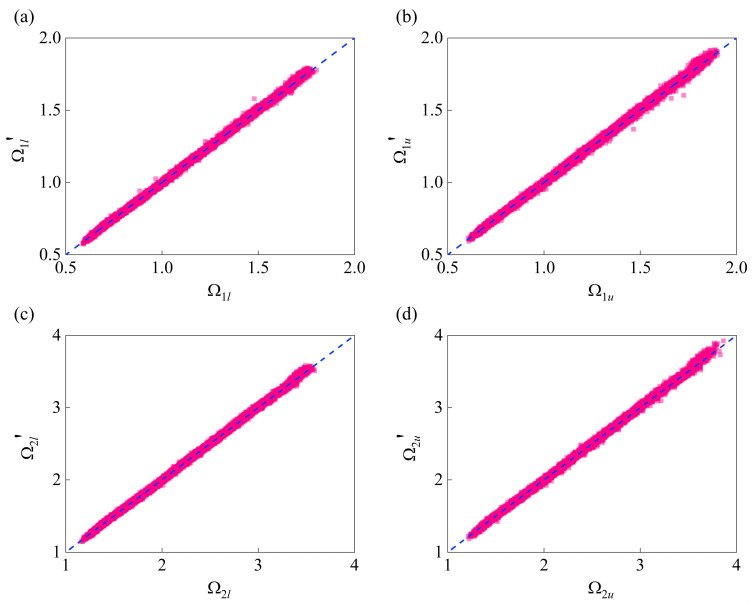
The predictions by the CVAE and ground truth of bounding frequencies over all the testing samples. The predictions are obtained based on the generated soft PnCs by the CVAE. The abscissa in the subfigures denotes the ground truth of bounding frequencies, and the ordinate axis denotes the prediction of bounding frequencies. In detail, (**a**,**b**) are the lower and upper boundaries of the first stop band, and (**c**,**d**) are the lower and upper boundaries of the second stop band. The blue dashed line reflects the physical meaning of “*y* = *x*”.

## Data Availability

The data presented in this study are available on request from the corresponding author due to privacy.

## Abbreviations

The following abbreviations are used in this manuscript:

PnCs	Phononic crystals
MMs	Metamaterials
BS	Bragg scattering
LR	Local resonance
1D	One-dimensional
FG	Functionally graded
2D	Two-dimensional
TO	Topology optimization
DL	Deep learning
ML	Machine learning
ANN	Artificial neural network
TNN	Tandem neural network
AE	Autoencoder
GAN	Generative adversarial network
MLP	Multilayer perceptron
DNN	Deep neural network
CVAE	Conditional variation autoencoder
CGAN	Conditional generative adversarial network
TMM	Transfer matrix method
FEM	Finite element method
MAE	Mean absolute error
NSGA-III	Nondominated Sorting Genetic Algorithm III
CC	Clamped condition

## Appendix A. Derivations of the Transfer Matrix Method

For *j*-th segment of the *n*-th unit cell, Equation (12) can be written as:

(A1) $$U(x_{3})=P_{j}^{n+}e^{ik_{j}x_{3}}+P_{j}^{n-}e^{-ik_{j}x_{3}}$$

where $P_{j}^{n+}$ and $P_{j}^{n-}$ are the displacement amplitudes in the *x*_3_ direction, while $k_{j}=\sqrt{\omega^{2}\rho /C_{3333}^{\left(j\right)}}$ is the wave vector of *j*-th segment, and *x*_3_ represents the position of the *j*-th segment in the unit cell, i.e., $x_{3}\in [h^{\left(1\right)}+h^{\left(2\right)}+\ldots +h^{\left(j-1\right)},h^{\left(1\right)}+h^{\left(2\right)}+\ldots +h^{\left(j\right)}]$ (*h*^(*j*)^ represents the length of the *j*-th segment). Therefore, the expressions of the displacement and stress on the *j*-th segment of the *n*-th unit cell can be formulated as:

(A2) $$U_{j}^{n}(x_{3})=P_{j}^{n+}e^{ik_{j}x_{3}}+P_{j}^{n-}e^{-ik_{j}x_{3}}$$

(A3) $$\sigma_{j}^{n}(x_{3})=C_{3333}^{\left(j\right)}ik_{j}(P_{j}^{n+}e^{ik_{j}x_{3}}-P_{j}^{n-}e^{-ik_{j}x_{3}})$$

According to the continuity conditions at the interface between the *j*-th and (*j* + 1)-th segments, the relations are required to satisfy:

(A4) $$u_{j}^{n}(h^{\left(1\right)}+h^{\left(2\right)}+\ldots +h^{\left(j\right)})=u_{j+1}^{n}(h^{\left(1\right)}+h^{\left(2\right)}+\ldots +h^{\left(j\right)})$$

(A5) $$\sigma_{j}^{n}(h^{\left(1\right)}+h^{\left(2\right)}+\ldots +h^{\left(j\right)})=\sigma_{j+1}^{n}(h^{\left(1\right)}+h^{\left(2\right)}+\ldots +h^{\left(j\right)})$$

By substituting Equations (A2) and (A3) into Equations (A4) and (A5), one can obtain that:

(A6) $$\Psi_{j+1}^{n}=T_{j}\Psi_{j}^{n}$$

where $\Psi_{j}^{n}=[P_{j}^{n+},\;P_{j}^{n-}]$ and **T***_j_* is the transfer matrix relating the *j*-th segment to the (*j* + 1)-th segment, which can be acquired by:

(A7) $$T_{j}={\left[\begin{array}{cc} e^{ik_{j+1}L^{\left(j\right)}} & e^{-ik_{j+1}L^{\left(j\right)}}\\ C_{3333}^{\left(j+1\right)}ik_{j+1}e^{ik_{j+1}L^{\left(j\right)}} & -C_{3333}^{\left(j+1\right)}ik_{j+1}e^{-ik_{j+1}L^{\left(j\right)}} \end{array}\right]}^{-1}\left[\begin{array}{cc} e^{ik_{j}L^{\left(j\right)}} & e^{-ik_{j}L^{\left(j\right)}}\\ C_{3333}^{\left(j\right)}ik_{j}e^{ik_{j}L^{\left(j\right)}} & -C_{3333}^{\left(j\right)}ik_{j}e^{-ik_{j}L^{\left(j\right)}} \end{array}\right]$$

here, *L*^(*j*)^ = *h*^(1)^+ *h*^(2)^ + …+ *h*^(*j*)^. Based on Equation (A6), the relationship between the first segment of the *n*-th unit cell and the first segment of the (*n* + 1)-th unit cell can be deduced as:

(A8) $$\Psi_{1}^{n+1}=T\Psi_{1}^{n}$$

where **T** = **T***_m_***T***_m_*_−1_‧‧‧**T**_1_. Additionally, the Bloch theory requires:

(A9) $$\Psi_{1}^{n+1}=e^{iqh}\Psi_{1}^{n}$$

Finally, the transfer matrix **T** can be obtained by associating Equation (A8) with Equation (A9).

## Appendix B. The Validation of the TMM Model

The material characteristics of phase *A* and *B* are given in Table A1, as illustrated from Ref. [88], each of which accounts for 0.5 of the entire domain. The normalized frequency is formulated by $\Omega =\omega H\sqrt{\rho_{A}/\mu_{A}}/(2\pi )$. Before performing band structure calculations, the adjustment on the equivalent modulus is illustrated in Figure A1 with respect to the varying prestress *σ*_0_. It is clearly seen that the tensional prestress can lower the equivalent modulus while the compression prestress is able to induce opposite effects.

**Table A1:** The material properties of phases *A* and *B*.

Material Phase	*ρ*/(kg/m^3^)	*μ*/(MPa)	*β*
*A*	1050	0.444	1
*B*	1260	0.888	2

Under fixed lateral equi-biaxial prestretch, the results are firstly computed by the TMM and FEM models separately in the cases of *σ*_0_ = −1 and *σ*_0_ = 1 and then compared with each other in Figure A2. The FEM simulation is conducted using the software COMSOL Multiphysics 6.0. As can be seen, whether for the soft PnC within tensile effects or compression effects, the band diagram acquired by the TMM agrees well with that by the FEM, which verifies the accuracy of the proposed TMM model.

## Appendix C. Supplement Materials of DL Models

The architectures of the forward model and the inverse model are illustrated in Figure A3a,b, respectively.

Further, the accuracy of DL-based models after training is examined. The average values of MAPE of both forward DL and inverse CVAE models over the test dataset (9540 test samples) are computed and provided in Table A2. Figure A4 and Figure A5 show the relation between the ground truth and the predictions via applying the forward model and CVAE model. It is worth noting that the predicted bounding frequencies in Figure A5 are obtained by the TMM model based on the soft PnCs generated by the CVAE. As can be seen, either the results from Table A2 or from Figure A4 to Figure A5 can demonstrate the superior capacity to generate feasible designs of soft PnCs via combining the forward DNN and the inverse CVAE.

**Table A2:** The average values of MAPE for estimating the accuracy of the forward and inverse models.

The Error Term	Forward DNN	CVAE
MAPE	0.23%	0.81%
